# Osteomalacia Is Not a Single Disease

**DOI:** 10.3390/ijms232314896

**Published:** 2022-11-28

**Authors:** Luisella Cianferotti

**Affiliations:** Bone Metabolic Diseases Unit, Department of Experimental and Biomedical Sciences, University Hospital of Florence, University of Florence, 50139 Florence, Italy; luisella.cianferotti@unifi.it

**Keywords:** phosphate, vitamin D, mineralization, hypophosphatasia, tumor-induced osteomalacia, rickets

## Abstract

Among bone-material qualities, mineralization is pivotal in conferring stiffness and toughness to the bone. Osteomalacia, a disease ensuing from inadequate mineralization of the skeleton, is caused by different processes leading to decreased available mineral (calcium and/or phosphate) or enzymatic alterations. Vitamin D deficiency, which remains the major cause of altered mineralization leading to inadequate intestinal calcium and phosphate absorption, may be also associated with other conditions primarily responsible for abnormal mineralization. Given the reality of widespread vitamin D inadequacy, a full biochemical assessment of mineral metabolism is always necessary to rule out or confirm other conditions. Both too-high or too-low serum alkaline phosphatase (ALP) levels are important for diagnosis. Osteomalacic syndrome is reversible, at least in part, by specific treatment. Osteomalacia and bone mineralization themselves constitute largely unexplored fields of research. The true prevalence of the different forms of osteomalacia and the recovery after proper therapy have yet to be determined in the real world. Although non-invasive techniques to assess bone mineralization are not available in clinical practice, the systematic assessment of bone quality could help in refining the diagnosis and guiding the treatment. This review summarizes what is known of osteomalacia recent therapeutic developments and highlights the future issues of research in this field.

## 1. Introduction

Osteomalacia is a metabolic bone disorder primarily characterized by altered mineralization [1].

Skeletal mineralization refers to the series of events leading to the deposition and maintenance of crystals of hydroxyapatite [Ca_10_(PO4)_6_(OH)_2_] into the bone matrix, in a proper pH environment and specific 3D structure [2,3,4]. The process of biomineralization confers the right stiffness to the complexity of the bone structure to resist multiple forces and to transform the musculoskeletal apparatus into a system of levers. Besides its structural function, mineralization has a key metabolic function, since it regulates bone cells’ microenvironment and constantly provides minerals to the extracellular milieu [3]. Mineralization is not homogeneous in the whole skeleton, since different types of bones mineralize differently according to their function.

This complex process is regulated by endocrine and paracrine factors and the availability of minerals, in particular calcium and phosphate. Biomineralization begins in prenatal life and continues throughout the life of living organisms, coexisting with both modeling and remodeling processes [4].

Osteomalacia, often referred to as the “disease of soft bones”, is the histological and clinical outcome of inadequate mineralization ensuing from various causes [1]. Thus, the metabolic bone disease osteomalacia is the final clinical and histological picture, sustained by different mechanisms, all leading to impaired skeletal tissue mineralization. While the development of morphologic alterations of rickets occurs only in developing skeletons along with modeling, osteomalacia may occur throughout life. Consequently, osteomalacia is not merely a disease of the adult bone, but osteomalacia-related abnormalities can be found also in the developing skeleton, so osteomalacia alterations may coexist with rachitic abnormalities. Moreover, individuals with rickets may become osteomalacic adults if not properly treated and/or the pathological cause persists through adulthood.

The first description of osteomalacia dates back to 1885, with an accurate histological description by Gustav Pommer, pointing out the distinctive features of rickets and osteomalacia with respect to other juvenile bone diseases (i.e., osteogenesis imperfecta, bone dysplasia, achondroplasia), osteoporosis, and osteitis fibrosa [5]. A decade later, Roentgen revealed, using X-rays, the peculiar characteristics of rickets and osteomalacia. At the beginning of the 20th century, along with the characterization of vitamin D and its roles in mineral metabolism, rickets and osteomalacia were classically linked to vitamin D deficiency and cured by vitamin D supplementation [5,6]. With the discovery of vitamin D-independent forms of rickets/osteomalacia, it is now clear that disorders of mineralization are not universally linked to vitamin D inadequacy, as further detailed in this paper.

While rickets-related bone alterations in the growing age are seldom observed nowadays in developed countries and are mainly diagnosed in the context of rare, genetically determined diseases, osteomalacia is a widespread, often underrated condition and can greatly hamper the quality and quantity of life of affected individuals [1].

While osteomalacia is a disease of bone metabolism characterized by impaired mineralization, osteoporosis is a systemic skeletal disease with decreased bone mass and altered bone micro- and microarchitecture, leading to brittle bones. Both conditions are characterized by altered bone quantity and quality and may lead to reduced bone strength, making them easier to break. When combined in a single patient, they concur independently from bone fragility [7].

The aim of this review is to describe the pathogenesis and the multiple causes of impaired mineralization and the histopathological, clinical, and radiological features of osteomalacia. A classification of the different forms of the disease and a feasible biochemical evaluation for differential diagnosis for everyday practice are proposed. The main forms of inherited and acquired osteomalacia will also be briefly described, along with non-specific and targeted therapeutic opportunities. Lastly, possible future developments in this field will be highlighted. 

## 2. Physiopathology of Defective Mineralization and Proposed Pathogenetic Classification of Mineralization Disorders

The deposition of organized hydroxyapatite crystals in the bone matrix is the key event of biomineralization. This dynamic process is regulated by multiple soluble paracrine and endocrine factors, an adequate amount of matrix deposited by osteoblasts, enzymes, availability of substrates, and a low concentration of mineralization inhibitors [3].

Classically, mineralization disorders were classified as “vitamin D-dependent” or “vitamin D-resistant” states, based on the response to treatment with vitamin D metabolites (cholecalciferol/vitamin D3, ergocalciferol/vitamin D2, 25 hydroxyvitamin D/25(OH)D/calcifediol, and 1,25 dihydroxyvitamin D/1,25(OH)_2_D/calcitriol) at usual doses [8] (Table 1A).

**Figure 1:**
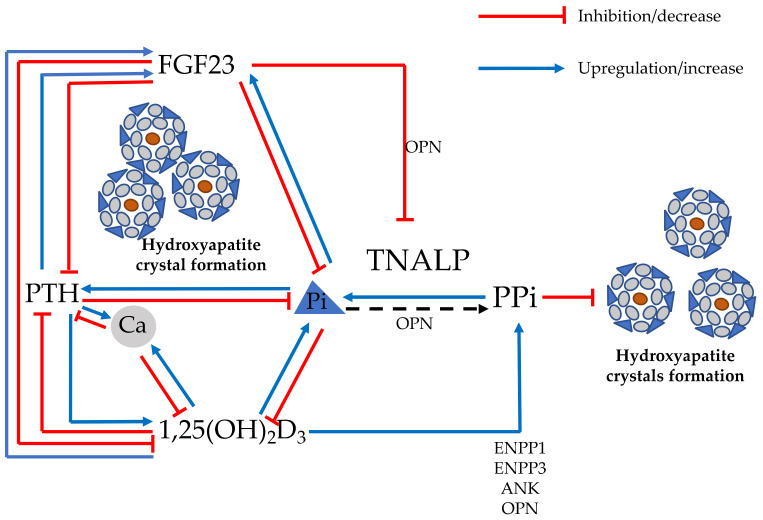
Graphic description of the endocrine and paracrine regulation of the biomineralization process in and around osteoblast and osteocytes and schematic representation of homeostasis feedbacks. Parathyroid hormone (PTH) and fibroblast growth factor 23 (FGF23) and phosphaturic hormones decrease serum Pi concentration, while 1,25-dihydroxy vitamin D_3_ [1,25(OH)_2_D_3_] increases serum Ca and Pi via active intestinal absorption through activation of the vitamin D receptor. These hormones are reciprocally regulated by negative feedback systems. Tissue non-specific alkaline phosphatase (TNALP) processes inorganic pyrophosphate (PPi), a major inhibitor of mineralization, turning it into Pi, which makes it available for hydroxyapatite crystal formation. At the same time, TNALP depohosphorylates and activates osteopontin (OPN), which, in turn, inhibits mineralization, increasing PPi. Other mineralization inhibitors include ectonucleotide pyrophosphatase phosphodiesterase 1 and 3 ectonucleotide pyrophosphatase phosphodiesterase 1 and 3 (ENPP1 and 3), which also increase the availability of PPi, similarly to OPN. Active vitamin D inhibits mineralization by increasing intracellular PPi via increasing expression of ENPP1 and 3 and OPN. Extracellular Pi per se inhibits mineralization, likely by increasing OPN. FGF23 decreases mineralization through direct inhibition of transcription of TNALP and through the upregulation of OPN.

The availability of substrates, i.e., calcium and phosphate present in the intestinal tract, which are actively absorbed by sufficient active vitamin D, i.e., [1,25(OH)_2_D], which in turn is produced in the kidney by parathyroid hormone (PTH) by stimulation of 1-alpha hydroxylase, is preliminary to optimizing the process of mineralization (Figure 1). These hormones and metabolites are interconnected and counter-regulated in a complex network to restore mineral homeostasis in response to physiologic or pathologic stimuli. Calcium and/or phosphate deficiency, excess of mineralization inhibitors, or hormonal excess/defects can lead to altered mineralization [9].

Prolonged and severe vitamin D deficiency leads to both calcium and phosphate inadequacy. Other forms of calcium and phosphate defectiveness, which can be at least in part vitamin D-dependent, might ensue from chronic malabsorption diseases, such as celiac disease or inflammatory bowel diseases, or be the medium- and long-term result of resective bariatric surgery or chronic liver and kidney disease [3,10,11]. Some drugs such as antiepileptics and glucocorticoids may disrupt vitamin D metabolism and, in some cases, also cause decreased calcium absorption and increased renal losses, contributing to hypomineralization. The rare genetically determined disorders vitamin D-dependent rickets (VDDR) types 1A and 1B, caused by mutation in genes encoding cytochrome P450 family 27 subfamily B member 1, i.e., 1alpha-hydroxylase (CYP27B1) or cytochrome P450 Family 2 Subfamily R Member 1, i.e., 25-hydroxylase (CYP2R1), are included in the vitamin D-dependent forms of altered mineralization since they are cured with specific analogs of vitamin D, such as 1alpha-hydroxylated forms of vitamin D or 25-hydroxylated vitamin D, respectively [1,12].

Additional types of osteomalacia are identified as vitamin D-resistant, since the replacement of vitamin D or its analogs does not correct the mineralization defect. Isolated phosphate deficiency per se can lead to profound alterations of mineralization. Serum phosphate levels are mainly regulated by the osteocyte-derived hormone fibroblast growth factor 23 (FGF23) [13]. FGF23 inhibits phosphate re-absorption by acting on the co-receptor FGFR1-Klotho and decreasing the expression and function of the sodium-dependent phosphate cotransporter in the renal proximal tubule (i.e., NAPT2a and NPT2c), so that an excess of FGF23 leads to hyperphosphaturia, phosphate wasting, and consequent hypophosphatemia [14]. Besides FGF23, PTH also regulates serum phosphate, similarly inhibiting renal phosphate re-absorption. FGF23 and PTH have opposite actions on renal 1 alpha-hydroxylase, thus inhibiting or enhancing the formation of di-hydroxyvvitamin D [1,25(OH)2D or calcitriol], respectively (Figure 1). Thus, FGF23 lowers serum phosphate directly and indirectly (by decreasing vitamin D-dependent intestinal absorption). 

FGF23 excess can be congenital, as determined by inherited, genetically determined disorders of phosphate metabolism, and can manifest in infancy with FGF23-dependent hypophosphatemic rickets, or acquired in adults in tumor-induced osteomalacia. An abnormal response in the renal apparatus to FGF23 action can be observed in acquired nephropathies such as Fanconi syndrome, also characterized by hypophosphatemia, along with tubular dysfunction [15].

Alkaline phosphatase (ALP), particularly its isoenzyme bone alkaline phosphatase (B-ALP), is an enzyme expressed in the membrane of differentiated early osteoblasts and in its secreted form is one of the serum markers of osteoblast function and bone formation. Its main role in the skeletal tissue is to convert inorganic pyrophosphate (PPi), a potent inhibitor of mineralization, into inorganic phosphate (Pi), making it available for nucleation and elongation of hydroxyapatite crystals disposed along the collagen fibrils [16]. Other osteoblast/osteocytes membrane enzymes, such as ectonucleotide pyrophosphatase/phosphodiesterase 1 (ENPP1), membrane transporter of PPi ANK, and other transmembrane proteins on osteoblasts such as PiT1 (sodium-phosphate transporter type III), contribute to the regulation of mineralization by modulating the extracellular concentration of PPi (Figure 1).

Mutations in the genes coding for the previously listed proteins may cause a mineralization disorder, usually diagnosed in infancy, at least in its severe forms [17]. Among these disorders, hypophosphatasia, the genetic metabolic disorder characterized by reduced activity of serum ALP, is one of the most notable [18]. Some individuals, often heterozygous for tissue non-specific alkaline phosphatase mutation (*TNSALP*), may manifest an abnormal mineralization only in adult age, mimicking acquired forms of mineralization defect, which have to be considered in the differential diagnosis [19]. These enzymes can be also the targets of certain drugs, known to interfere with mineralization, such as bisphosphonates. Genetically determined alterations in the response apparatus to active vitamin D, i.e., vitamin D receptor, VDR, or a hormone-response element protein, can lead to vitamin D-dependent rickets type 2A and 2B, respectively (VDDR2). This is a misleading nomenclature, since these forms are typically “resistant” to all forms of vitamin D analogs, clearly including 1,25(OH)2D [20].

From a clinical point of view, taking advantage of the additional possibilities of biochemical and genetic diagnosis, a classification based on the time-of-onset of the disease (early- versus late-onset) or based on the pathogenesis (congenital versus acquired) is therefore proposed (Table 1B,C). Congenital disorders of mineralization mainly appear as early-onset diseases and are primarily characterized by rickets, even if features of osteomalacia will also be manifest in young bones. Acquired forms are mainly late-onset and are primarily characterized by clinical signs and symptoms of osteomalacia in adults [1].

## 3. Histopathological Features of Osteomalacia

Although osteomalacia can be the outcome of several pathogenetic mechanisms, it is characterized by common histological features. Indeed, the historical definition of osteomalacia is histopathological. The histological signs of osteomalacia appear if the cause leading to impaired mineralization persists over time [21].

The sequence bone formation–mineralization–resorption is the coordinated process taking place at every remodeling unit in the mature skeletal tissue, in both young and adults, requiring and providing the minerals from and to the extracellular compartment, respectively.

Classically referred to vitamin D-dependent forms, a grading of osteomalacia was attributed to the evolution of bone alterations as histologically revealed [22]. In the first stages of the mineral metabolic disorder, the key players in the mineralization process, i.e., hormones and enzymes, adapt in order to provide the skeleton with a sufficient amount of minerals to form and replace previously resorbed mineralized matrix and correct the metabolic abnormalities (i.e., secondary hyperparathyroidism in response to vitamin D deficiency). At this stage, no histopathological aberrations are detected in the skeleton, but general symptoms may be present due to systemic metabolic abnormality (stage I, or pre-osteomalacia). If the metabolic alteration persists and cannot be reverted by metabolic adaptations, crystals of hydroxyapatite do not form and matrix cannot be mineralized [22]. Osteoclasts per se are not able to reabsorb the unmineralized matrix, which continues to be produced by osteoblasts and therefore accumulates at every remodeling cycle and at each remodeling surface. The increased amount of osteoid is the hallmark of all forms of osteomalacia, causing a softening of the bone tissue, with decreased stiffness and resistance. The progressive accumulation of osteoid, together with some residual mineralization, is typical of stage II. Stage III is the complete cessation of mineralization with a considerable amount of osteoid, which corresponds to the traditional histological picture of overt osteomalacia [5]. Histomorphometrically, bone formation is abolished, as demonstrated by the absent uptake of tetracycline, with decreased bone volume per tissue volume (BV/TV), increased osteoid thickness (width of the osteoid seams >15 μm), volume, and surface. The absent mineralization, as demonstrated by the long mineralization lag time (>100 days), is typical of osteomalacia at histomorphometry and distinguishes this condition from other bone diseases (i.e., hyperthyroidism, hyperparathyroidism, Paget disease of the bone), which may be also characterized by an increased amount of osteoid ensuing from increased bone turnover [22,23].

**Figure 2:**
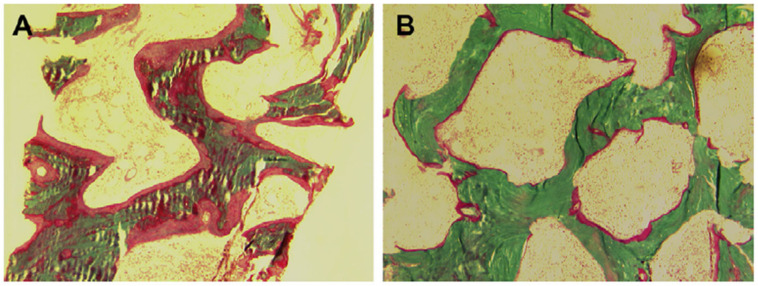
Reversibility of osteomalacic histopathological features after proper correction of vitamin D deficiency: the increased amount of osteoid (in red) deposited over poorly mineralized trabecular bone (green) (panel (**A**)) is decreased in surface and thickness (panel (**B**)) (reproduced with permission from ref. [5]).

Ostemalacic lesions can be variable in number and size at histological examination. The amount of the osteoid is usually proportional to the degree and duration of the abnormality of mineral metabolism, with thicker osteoid patches being the result of long-standing disorders. While modeling defects of mineralization (i.e., rickets) may cause permanent skeletal alteration even if mineral homeostasis is restored (i.e., bone deformities in rickets), the histopathological features of osteomalacia can be reversible at least in part once their metabolic abnormalities have been corrected [5] (Figure 2). Nonetheless, as a result of secondary hyperparathyroidism, endocortical bone is also resorbed, leading to irreversible cortical thinning and increased fracture risk [24].

## 4. Clinical and Radiological Manifestations

The general clinical characteristics of osteomalacia, sometimes referred to as osteomalacic syndrome, are common among disorders ensuing from different causes [1]. The classical definition of osteomalacia as the “softening of the bone tissue” is somewhat misleading, since bone deformities, which may be inferred from this definition, are not typical of an osteomalacic skeleton, except in a few special circumstances, but usually represent the consequence of rickets that were not properly treated in a growing individual. If rickets-related bone deformities are visible (bowed legs, frontal bossing, chest irregularities, and short stature, along with a history of multiple osteotomies to straighten up legs), the diagnosis should be oriented towards congenital/early-onset disorders [1]. These latter individuals may be prone to also develop osteoarthritic lesions at the knees and the hips deriving from altered axial alignment [25].

Signs and symptoms in mild/early disease can be vague and non-specific, while overt osteomalacia is usually symptomatic with distinctive musculoskeletal features. In this latter case, affected patients may present with generalized bone pain and tenderness, which may often lead to overuse and/or abuse of anti-inflammatory medications. The dull, diffuse bone ache in osteomalacia is due to the hydration of unmineralized matrix at the periosteum, which elicits peripheral nociceptors. The pain is also present at rest, but it becomes worse on weight bearing and standing. Skeletal tenderness refers to the ability to evoke pain with pressure over superficial bones, such as the sternum, anterior tibias, wrists, and ribs. The non-specificity of these signs and symptoms, which can also be related to rheumatologic disorders (polymyalgia, fibromyalgia, and ankylosing spondylitis), may significantly delay the diagnosis and appropriate treatment [1,5].

When a severe abnormality in mineral metabolism occurs in a relatively short period of time, such as hypophosphatemia in tumor-induced osteomalacia due to an acquired excess of FGF23, skeletal deformities might ensue, resembling a very high-turnover disease, such as historical severe forms of untreated primary hyperparathyroidism, which may constitute a phenocopy of this disease. These patients may show deformity of the thoracic cage with a pigeon chest and increased spinal curvature associated with decreased height [26].

**Figure 3:**
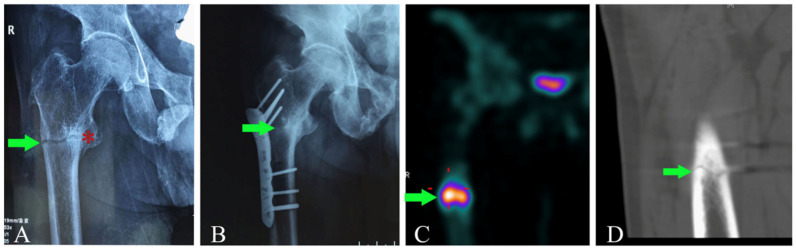
(**A**): Transverse fracture line of the proximal femur, with callus formation (star) as well as cortex breakage (arrow) simultaneously. (**B**): Successful healing of the primary pseudofracture of the proximal femur (arrow). (**C**): High uptake of 99mTc-MDP beneath the osteosynthesis plate, indicating a new pseudofracture of the proximal femur (arrow). (**D**): The transverse fracture line (arrow) revealed by computed tomography scanning (as reproduced with permission from ref. [27]).

Since the unmineralized bone is more fragile, spontaneous cortical bone cracks, also referred to as pseudofractures, may occur. Since they are the clinical outcome of a systemic disease, the pseudofractures (also referred to as Milkman lines, Looser zones, or insufficiency/stress fractures) appear as a radiolucent, transverse bands. The pseudofractures are more common in the main load-bearing bones such as the proximal and diaphyseal femur (subtrochanteric region), pelvis, and metatarsals and are often symmetric and bilateral, even if they can occur asynchronously in time since they are the expression of a systemic disease (Figure 3). These lesions might be preceded by localized bone pain. For the above reasons, it is advisable to examine the contralateral bone once a pseudofracture is diagnosed and identified.

Besides pseudofractures, low-energy trauma fractures of the flat bones such as the sternum and ribs may take place, constituting a typical sign of osteomalacia in the adult skeleton. Rachitic rosary, a typical sign of rickets, related to the expansion of the anterior rib ends at the costochondral junctions, may be also observed in adults with tumor-induced osteomalacia. Osteomalacic vertebrae are often referred to as “codfish vertebrae” in the spine radiography, which refers to a particular smooth biconcave deformity of the vertebral bodies distinguished from the classic fragility fractures related to osteoporosis. The upper and lower borders of osteomalacic vertebrae are symmetrically deformed. Given the metabolic nature of the disease, contiguous vertebrae usually display similar abnormal biconcaval shapes [28]. Conversely, osteoporotic vertebral fractures, even when there are multiple in the same patient, are heterogenous in shape deformity, so in this case, the loss of self-similarity between adjacent vertebrae is a specific radiological sign. Moreover, cortical discontinuities are often present, and vertical trabeculations conferring striated appearance to the vertebrae are often present [29]. The Genant’s visual semiquantitative method to define vertebral fractures in lateral radiographs, based both on the vertebral shape and on percentual height loss involving the anterior, posterior, and/or middle vertebral body, could theoretically be applied also to osteomalacic vertebrae, since it provides a grading of column bone deformities (i.e., spinal deformity index) independently of bone disease, although no specific statements nor systematic assessments have been issued/employed in osteomalacia [30].

The involvement of the skeletal muscle, once named “osteomalacic myopathy”, is characterized by a moderate-to-severe proximal muscle weakness, mainly involving the shoulder and pelvic girdle, hampering walking with a consequent waddling gait and/or impaired standing up from sitting position. The decreased number and diameter of the fast-twitch type II fibers, the first to be recruited to avoid falling or for major muscle efforts, has been demonstrated [31].

Bone dual energy X-ray absorptiometry (DXA) scan usually shows decreased bone mineral density (BMD) because of decreased mineralization. Indeed, differential diagnosis with other diseases characterized by low BMD such as osteoporosis, renal osteodystrophy, and primary hyperparathyroidism with or without osteitis fibrosa cystica, has to be made. The usual mistake of interpreting low BMD results just as “osteoporosis”, which is a disorder primarily characterized by altered bone microarchitecture, may lead to inappropriate diagnosis and treatment [32]. Conversely, osteomalacia is not always associated with low BMD, since adults with XLH may display higher BMD values due to enthesopathy (lumbar spine BMD) or independently from that (hip BMD) [15].

The decrease in BMD due to a mineralization defect is at least in part reversible with specific remineralization treatment, retrospectively indicating the nature of the bone disease [1]. Backscattered electron imaging is a technique that can be applied to bone biopsies to assess mineralization. However, in the research setting, this technique could be useful in distinguishing disorders of mineralization such as osteomalacia from other diseases such as osteoporosis, as further detailed next in this paper (Section 8).

Since osteomalacia is a metabolic condition, bone scintigraphy may reveal multiple areas with increased uptake of the radiolabeled ^99m^tecnetium-diphosphonate, reflecting the abnormal osteoblast activity and/or recent/incipient pseudofractures [33]. In this respect, the bony alterations observed at physical examination and/or on X-rays may appear with enhanced uptake. This exam may also help in distinguishing osteomalacia from a Paget disease of bone. These abnormalities may disappear with appropriate remineralizing therapy and normalization of bone metabolism.

Dental status should always be checked. While osteomalacia is not associated with the development of dental defects per se, a history of multiple cavities, premature tooth loss, or spontaneous avulsions, dentinal alterations may suggest a congenital/genetically determined disorder (hypophosphatemic rickets/osteomalacia, hypophosphatasia), since dental abnormalities are common in these forms, thus helping in the differential diagnosis [34].

## 5. Biochemical Features and Differential Diagnosis

The clinical and/or radiological suspicion of a mineralization defect should guide the biochemical diagnostic workout to confirm the diagnosis and to establish the cause.

When evaluating disorders of mineralization, a key analyte to be determined is ALP, in order to make diagnosis, estimate disease severity, and monitor the effect of therapy [35]. ALP is the serological marker of overt osteomalacia, especially when it is persistently elevated. ALP levels usually reflect the increased osteoblastic activity occurring in osteomalacia and are proportional to the amount of osteoid observed in the histological exam [22]. In stage I and II osteomalacia (recent-onset, mild, localized osteomalacia), ALP levels can be normal or in the upper normal range.

ALP levels are low in hypophosphatasia, which can be included in the diseases of impaired mineralization since even in this condition an osteoid excess is present. Assessment of serum vitamin B6 piridossal-5-phosphate (PLP) and urinary phosphoetanolamine (PEA), high in this disease, is mandatory to confirm the diagnosis [36].

High ALP levels can occasionally be detected in routine biochemical testing and impose a differential diagnosis with liver diseases, Paget disease of bone, primary hyperparathyroidism, renal osteodystrophy, recent fracture/fractures, and other skeletal diseases with high bone turnover and possible bone deformities. Even low ALP levels pose problems of differential diagnosis since they can be transiently or precipitously low due to different conditions [37].

The determination of serum (corrected) calcium, phosphate, PTH, 25(OH)D is mandatory. More rarely, 1,25(OH)2D and FGF23 can be determined in the case of specific rare diseases [1]. Genetic tests could be undertaken to confirm the diagnosis in the case of genetically determined forms. The main biochemical traits of the various forms of osteomalacia are illustrated in Table 2.

Serum levels of 25(OH)D, the marker of vitamin D status, will help in refining the diagnosis. Levels less than 20 ng/ml (i.e.50 nmol/L) indicate vitamin D deficiency, which is severe if less than 10 ng/ml (i.e., 25 nmol/L). Vitamin D deficiency, which is common in the general population particularly at northern latitudes for most of the year, does not exclude other causes of osteomalacia that always have to be considered, even in the presence of low or exceedingly low levels of 25(OH)D. Thus, vitamin D deficiency, especially if long-standing, can be the main pathogenetic factor or, at least, a co-factor in osteomalacia. Secondary hyperparathyroidism, which occurs in the face of poor calcium absorption due to low vitamin D, guarantees the maintenance of serum calcium in the normal or low-normal range, even in the face of severe forms of vitamin D inadequacy. Overt hypocalcemia and hypophosphatemia may be observed in the so-called “vitamin D-dependent” genetically determined rickets/osteomalacia because of the lack of the action of the biologically active hormone calcitriol.

The finding of levels of serum phosphate in the low-normal or decreased range is relatively common in early-onset rickets/osteomalacia [38].

When hypophosphatemia is the only serological finding, a disorder of phosphate metabolism can be inferred. In order to distinguish between FGF23-dependent and FGF23-independent hypophosphatemia, the renal tubular reabsorption of phosphate has to be calculated by Tm_p_/GFR (i.e., the maximum rate of tubular phosphate reabsorption divided by the glomerular filtration rate, as assessed by serum and urinary phosphate and creatinine determined in fasting urine and serum samples) [15]. If Tm_p_/GFR is low, phosphate wasting can be hypothesized, and an FGF23 excess can be inferred. Tm_p_/GFR is also mildly decreased in disorders due to vitamin D deficiency or calcium depletion, given the secondary hyperparathyroidism causing increased phosphate excretion. Conversely, a decreased Tm_p_/GFR points towards phosphate depletion due to other causes, e.g., tubulopathies. Investigating potential urinary losses (amino acids, bicarbonates, etc.) could help in confirming this diagnosis. If no other alterations are found, serum FGF23 measurement should be undertaken to direct the diagnosis of FGF23-independent hypophosphatemia [39,40].

The assessment of 1,25(OH)2D helps in the differential diagnosis of FGF23-dependent conditions since an excess of FGF23 inhibits renal 1 alpha-hydroxylase and leads to levels of 1,25(OH)2D, often in the low-normal range or less frequently below. Low levels of the biologically active vitamin D may also be low in VDDR1 or very high in diseases with end-organ resistance to calcitriol (VDDR2) [20].

The measurement of FGF23, when available, is important in the differential diagnosis of osteomalacia [41]. While levels of FG23 are within the normal or low-normal range in the majority of osteomalacia-related diseases, they are inappropriately high by definition in FGF23-dependent disorders [42].

When evaluating young subjects, specific pediatric reference ranges must be considered, since the concentration of minerals (serum phosphate, calcium) and enzymes (ALP) is shifted towards higher values, so that results confronted with an “adult” reference range can be misinterpreted in children/adolescents.

## 6. More Frequent Forms of Osteomalacia in Clinical Practice

### 6.1. Acquired Osteomalacia

Acquired osteomalacia can occur at all ages.

Despite the widespread use of vitamin D supplements, vitamin D deficiency-related osteomalacia is still common in particular categories of patients, such as both community-dwelling and institutionalized elders [43]. A large spectrum of conditions can be associated with vitamin D deficiency. Insufficient exposure to sunlight, inadequate dietary intake, cultural habits (use of veil or hijab), use of sunscreens, morbid obesity, malabsorption syndromes caused by different gastrointestinal disorders, and aging itself with decreased cutaneous production of vitamin D may contribute to decreased levels of serum 25(OH)D. Kidney and chronic liver diseases or drugs interfering with hydroxylases might alter the production of semi-active and active metabolites of the vitamin (i.e., 25(OH)D and 1,25(OH)2D, respectively) [1,8].

Vitamin D-dependent osteomalacia (and, of course, rickets) is rare in young individuals in industrialized countries, but it is still epidemic in some regions of the world and in particular groups of people (e.g., immigrants from South East Asia, Africa, and Middle East) because of cultural habits (i.e., covering skin) and/or skin pigmentation [44].

Malabsorption syndromes (celiac disease), inflammatory bowel diseases, generic malnutrition (common in elderly people), and pancreatic dysfunction may lead to chronic impaired calcium and phosphate intestinal absorption. An unbalanced vegan diet is often associated with nutritional deficiencies of various degrees and may be responsible for even severe forms of osteomalacia in both young people and adults [45]. Iatrogenic malabsorption resulting from surgical procedures for rapid weight loss (i.e., gastric bypass) is much more frequent today than in the past [10]. Acquired severe osteomalacia is observed today, mainly as a result of extended surgical resections, which have now been replaced by more conservative surgical interventions. If the risk of gross deficiencies of macro-nutrients is less common after these latter procedures, the deficiency of micronutrients is still common, leading to bone consequences in the long term (insufficiency fractures and bone and muscle pain) [46].

In vitro and vivo evidence point towards environmental contaminants as a potential cause or concurrent cause of bone diseases [47]. Persistent exposure to cadmium and aluminum overuse/abuse have been linked to alteration in bone cells and in mineralization [48,49]. Indeed, a vast majority of people living in a cadmium-polluted area develop osteomalacia, as revealed in autopsies [49].

Tumor-induced osteomalacia (TIO) is a paraneoplastic syndrome characterized by severe, rapidly progressing demineralization caused by usually small mesenchymal tumors secreting an excess of FGF23, leading to hypophosphatemia due to phosphate wasting [27]. It usually occurs in the fourth and fifth decades of life. Affected patients, previously in apparently good health, quickly present worsening diffuse bone pain, muscle weakness, and gait abnormalities, often associated with bone fragility (i.e., multiple insufficiency fractures), height loss, chest wall deformities, and generalized debilitated status [50,51]. Delay in diagnosis and in tumor localization is common. Bone fragility and reduction in BMD are often misinterpreted as osteoporosis, leading to inappropriate antiresorptive treatments prescribed before proper diagnosis. Hypophosphatemia, decreased Tm_p_/GFR, and high FGF23 levels are the characteristic biochemical features. The localization of the phosphaturic mesenchymal tumors can be challenging. In this respect, functional imaging (octreotide scintigraphy and Fluoro18 deoxyglucose positron emission tomography, Gallium68 DOTATATE, DOTATOC or DOTANOC imaging) can be employed in order to identify a suspicious hypermetbolic area that can subsequently be analyzed by focused anatomical imaging (magnetic resonance and/or computer tomography) [52]. Since, although rarely, cutaneous lesions can be responsible for TIO, a full examination of the skin is also necessary in the case of negative imaging. In TIO, surgical treatment is the therapy of choice once the tumor has been localized, and it usually reverts to a normal clinical picture.

### 6.2. Inherited Osteomalacia

Inherited osteomalacia is usually overcome and preceded by manifestations of rickets.

The X-linked dominant hypophosphatemic rickets/osteomalacia (XLH) is the most frequent form (prevalence 1/20,000). In XLH, the haploinsufficiency of the phosphate-regulating endopeptidase homolog, X-linked gene (*PHEX*) leads to an excess of the phosphaturic FGF23 [53,54]. Signs of osteomalacia are more evident in adults and coexist with deformity of weight-bearing limbs and dental abnormalities as has occurred in childhood. Bone and joint pain, joint stiffness, muscle pain and weakness, and abnormal gait decrease the quality of life in adult patients. Recently, a study in an animal model of this disorder showed that subchondral osteomalacia is responsible for enthesopathy, a painful abnormal mineralization of the fibrocartilagineous tendinous insertion site, which contributes to the clinical picture in adults and greatly impairs physical function [55]. Other even rarer genetically determined diseases caused by an excess of FGF23 and a similar clinical picture are listed in Table 3.

Another disorder manifesting as osteomalacia in adults is hypophosphatasia. While recessive mutations of the *TNSALP* gene give rise to a more severe diseases manifesting early in childhood, adult hypophosphatasia is a genetically determined disorder of mineralization, usually determined by haploinsufficiency of *TNSALP*, in which osteomalacia in a part of a more complex clinical picture, characterized by multiple insufficiency fractures, chondrocalcinosis, osteoarthropathy, and dental abnormalities (early loss of primary and secondary teeth). The real prevalence in adults is not known today, but it is supposed that is greatly underestimated, since this disorder can be often misinterpreted as a rheumatologic disease and not properly diagnosed [56].

## 7. Prevention and Treatment

Once the etiology has been established, specific treatment has to be initiated.

When vitamin D deficiency is the major cause or contribute to the development of osteomalacia, supplementation with adequate amounts of biologically inactive vitamin D analogs (i.e., cholecalciferol, or vitamin D3, or calcifediol [25(OH)D]), restore calcium and phosphate homeostasis, correct secondary hyperparathyroidism, and revert to normal, at least in part, the radiographic and histological abnormalities relatively quickly, depending on the administered dosage [5]. While there is general consensus among international societies that values lower than 10 ng/ml of serum 25(OH)D must be avoided at all ages to prevent rickets/osteomalacia, disagreement exists on the minimum desirable 25(OH)D concentration to be achieved during treatment [43,57,58]. Accordingly, recommended intake of vitamin D varies among different guidelines (800–1000 IU to higher dosages). According to a global consensus, for the prevention and treatment of nutritional rickets (and osteomalacia), cholecalciferol may be administered in loading dose, followed by a maintenance regimen in the long term, with daily, weekly, or monthly doses. The oral administration of cholecalciferol always has to be preferred because of better bioavailability with respect to the parenteral administration, which has to be recommended just in cases of malabsorption syndrome. When osteomalacia is associated with osteoporosis, the correction of the mineralization defect should precede antiresorptive therapy. In this respect, if an osteomalacic condition has to be corrected in a short time, calcidiol, which is more potent than cholecalciferol in terms of targeting the targeted levels of serum 25(OH)D (>30 ng/mL according to most guidelines for people with bone disease) and is approved to treat osteomalacia, is a good option [57,59,60,61,62]. The daily intake of minerals (calcium, phosphate, and magnesium) has also to be estimated by specific questionnaires and adequately integrated when proved to be deficient [63]. While diet is rarely poor in phosphorus content, the consumption of foods rich in calcium often has to be encouraged. When diet cannot be modified, calcium supplements (i.e., calcium carbonate or citrate) can be administered. Calcium carbonate (40% calcium per pill) should be taken preferably with meals, since an acidic gastric environment is needed to dissolve and absorb the tablets. Calcium citrate (20% calcium per pill) can be absorbed on an empty stomach or during iatrogenic modification of gastric pH (i.e., chronic therapy with proton pump inhibitors). Its better bioavailability decreases side effects (i.e., dyspepsia and constipation) commonly reported with calcium carbonate [64]. In cases of severe osteomalacia due to malabsorption syndromes, such as in extensive intestinal resection, high amounts of calcium, together with the biologically active vitamin D, calcitriol, have to be administered in order to try to surmount the serious impairment in the active absorption of minerals and vitamins.

In congenital or acquired diseases in which 25-hydroxylation or 1alpha-hydroxylation is lacking (VDDR1 or drugs), a specific vitamin D metabolite (calcidiol or calcitriol) is the therapy of choice. In end-organ resistance to calcitriol (VDDR2), large amounts of i.v. and oral calcium and oral calcitriol are needed in order to overcome the VDR multifunction.

In inherited hypophosphatemic osteomalacia, the standard-of-care practice is the administration of phosphate salts together with calcitriol, to avoid secondary hyperparathyroidism. While this treatment is mandatory in children to avoid rachitic deformities, the administration of phosphate salts, which are poorly tolerated and associated with important short- and long-term side effects, has been questioned in adults [65]. Nonetheless, in these hypophosphatemic patients, the correction of osteomalacia is needed, especially in some conditions, in order to optimize mineralization. Burosumab (KRN23) is a human monoclonal antibody against FGF23, which is successfully employed in FGF23-dependent hypophosphatemic disorders (i.e., XLH and TIO) and approved in Europe for the treatment of these diseases [66,67,68]. In particular, in TIO burosumab is recommended when tumors “cannot be curatively resected or localized” [66].

Asfotase alfa is the replacement enzyme approved for the treatment of hypophosphatasia in both children and adults [69,70,71].

## 8. Research Gaps and Potential Development in the Field

To date, the diagnosis of osteomalacia is mainly clinical and biochemical, given the great limitations in defining osteomalacia by the widely available DXA. The systematic assessment of bone quality in osteomalacia, in order to find specific patterns of this disease, would help in refining the radiological diagnosis. Indeed, the trabecular bone score (TBS), an index to infer bone microarchitecture in DXA images, might help in differentiating osteomalacia from osteoporosis [72]. Indeed, TBS and high-resolution peripheral quantitative computed tomography (HRpQCT) have started to be employed to better analyze bone impairment in TIO, in which they are correlated and demonstrated to be useful in assessing bone impairment versus DXA [73].

**Figure 4:**
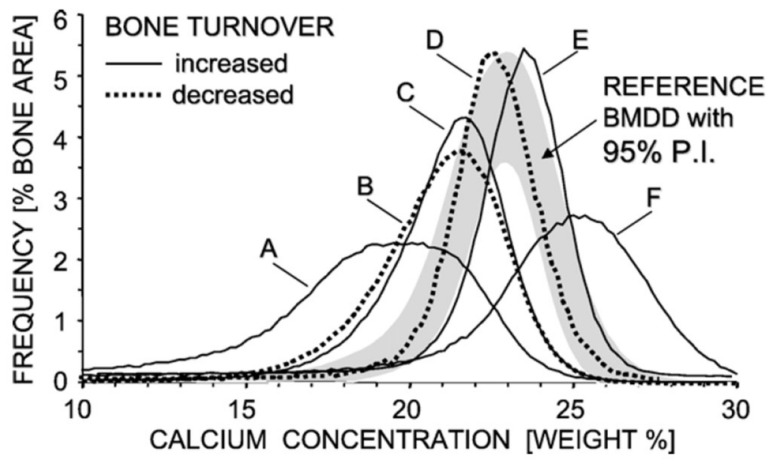
Bone mineralization density distribution (BMDD) of healthy and diseased or treated bone (transiliac biopsies). (**A**): Osteomalacia (celiac disease); (**B**): idiopathic osteoporosis; (**C**): postmenopausal osteoporosis; (**D**): postemenopausal osteoporosis treated with bisphosphonates; (**E**): osteogenesis imperfecta type I; (**F**) postmenopausal osteoporosis treated with sodium fluoride (reproduced with permission from ref. [32].

The application of old techniques in clinical practice such as scanning electron microscopy (SEM) to undecalcified bone biopsies is useful to analyze mineralization and establish the bone mineralization density distribution (BMDD) but has been progressively abandoned in clinical practice being an invasive technique [32]. The employment of backscattered electrons (BSEs), high-energy electrons produced by the elastic scattering of the primary beam electrons with the interaction with the examined material, is exploited by quantitative backscattered electron imaging (qBEI) to determine bone-mineralization density distribution [32] (Figure 4). The homogeneity/heterogeneity of bone mineralization, as represented by a narrower or larger curve, respectively, and the frequency of the different calcium amounts reflect mineralization quality. In this context, osteomalacia is represented by a larger and blunted curve, indicating low amounts of mineral and greater mineralization heterogeneity. This technique, still limited to very restricted research settings, could be exploited more in the future to assess mineralization and better characterize the various disorders and the responses to treatment.

More specific biomarkers of the diseases (microRNAs, extracellular vesicles) could be studied in order to find specific patterns in osteomalacia [74]. 

In addition, the medium- and long-term outcomes of specific therapies (e.g., burosumab and asfotase alfa) still need be assessed in real-life long-term studies.

## 9. Conclusions

Osteomalacia is a broad term that indicates inadequate mineralization of the skeleton, which may be caused by several defects in mineral metabolism. Osteomalacia can be genetically determined, so it is generally diagnosed in infancy because it is generally preceded by rickets. At the same time, osteomalacia can be a subtle disease in adults, since it resembles other disorders, so multiple phenocopies of this disease delay the diagnosis and, therefore, the commencement of specific treatment. Osteomalacia and bone mineralization itself, remain largely unexplored fields of research. The real prevalence of the different forms of osteomalacia and the specific recovery are yet to be determined in the real world. Finding specific biomarkers of these diseases and specific features using bone quality assessment and specific mineralization analysis (qBEI) will help in refining the diagnosis and quickly address the quality of life and disease burden in these patients.

## Figures and Tables

**Table 1:** Classification of osteomalacia based on: response to treatment with vitamin D metabolites (1A), time-of-onset (1B), and pathogenesis (1C).

**1A—Classification of osteomalacia based on response to treatment with vitamin D metabolites.**
**Vitamin D-Sensitive Osteomalacia**
**Vitamin D deficiency** **Malabsorption syndromes** (e.g., celiac disease, bariatric surgery) **Malnutrition** (e.g., unbalanced vegan diet) **Chronic liver diseases** **Chronic kidney diseases** **Drugs interfering with vitamin D metabolism** **Vitamin D-dependent rickets** - VDDR1A - VDDR1B
**Vitamin D-Resistant Osteomalacia**
**Vitamin D-dependent rickets** - VDDR2A - VDDR2B **Hypophosphatemic disorders** - FGF23-related - renal tubular dysfunction (Fanconi syndrome) - alcohol abuse **Impaired mineralization-related enzyme activity** - drugs interfering with mineralization process - metal chronic exposure/intoxication - hypophosphatasia
**1B—Classification of osteomalacia based on the time-of-onset.**
**Early-onset Rickets/Osteomalacia**
**Genetically determined hypophosphatemia** - X-linked dominant hypophosphatemic rickets/osteomalacia (XLH) - Autosomal dominant hypophosphatemic rickets/osteomalacia (ADRH1 and ADRH2) - Autosomal recessive hypophosphatemic rickets/osteomalacia (ARHR1 and 2) - Hypophosphatemic disease with dental anomalies and ectopic calcification - Hereditary hypophosphatemic rickets with hypercalciuria (HHRH) - Hereditary Fanconi renotubular syndrome (FRTS) - Dent’s disease (DENT) - McCune–Albright syndrome/fibrous dysplasia (MAS) - Cutaneous skeletal hypophosphatemia syndrome (CSHC; e.g., linear sebaceous nevus syndrome) **Vitamin D-dependent rickets (VDDR)** - VDDR1A - VDDR1B - VDDR2A - VDDR2B
**Early- or Late-onset Rickets/osteomalacia**
**FGF23-dependent** - Tumor-induced rickets/osteomalacia (TIO) - Acquired Fanconi syndrome - Drug nephrotoxicity - Saccharated ferric oxide or iron polymaltose therapy - Alcohol abuse **FGF23-independent** - Vitamin D deficiency - Malabsorption syndromes (celiac disease, bariatric surgery) - Malnutrition (vegan diet) - Chronic liver diseases - Chronic kidney diseases - Drugs interfering with vitamin D metabolism - Metal chronic exposure/intoxication - Hypophosphatasia
**1C—Classification of osteomalacia based on the pathogenesis.**
**Congenital Osteomalacia**
**Genetically determined hypophosphatemia** - X-linked dominant hypophosphatemic rickets/osteomalacia (XLH) - Autosomal dominant hypophosphatemic rickets/osteomalacia (ADRH1 and ADRH2) - Hypophosphatemic disease with dental anomalies and ectopic calcification - Hereditary hypophosphatemic rickets with hypercalciuria (HHRH) - Hereditary Fanconi renotubular syndrome (FRTS) - Dent’s disease (DENT) - McCune–Albright syndrome/fibrous dysplasia (MAS) - Cutaneous skeletal hypophosphatemia syndrome (CSHC; e.g., linear sebaceous nevus syndrome) **Vitamin D-dependent rickets (VDDR)** - VDDR1A - VDDR1B - VDDR2A - VDDR2B **Hypophosphatasia**
**Acquired Osteomalacia**
**FGF23-independent** - Vitamin D deficiency - Malabsorption syndromes (celiac disease, bariatric surgery) - Malnutrition (e.g., vegan diet) - Chronic liver diseases - Chronic kidney diseases - Drugs interfering with vitamin D metabolism - Metal chronic exposure/intoxication **FGF23-dependent** - Tumor-induced rickets/osteomalacia (TIO) - Acquired Fanconi syndrome - Drug nephrotoxicity - Saccharated ferric oxide or iron polymaltose therapy - Alcohol abuse

**Table 2:** Biochemical features (serum and urinary parameters) with differential diagnosis of the various forms of osteomalacia (=: usually in the normal range; ↓: usually decreased; ↑: usually increased; =↓: usually in the normal range or decreased; =↑: usually in the normal range or increased; ↓↓: markedly decreased; ↑↑: markedly increased; =↑↓: normal, increased or decreased).

	ALP	Pi	TmP/GFR	Ca	PTH	25(OH)D	1,25(OH)_2_D	FGF23
**Vitamin D deficiency**	=↑	=↓	=↑	=↓	=↑	↓↓	=↑↓	=↓
**Malabsorption syndromes, other nutritional deficiencies**	=↑	=↓	=↑	=↓	↑/↑↑	↓	=↑↓	=↓
**Vitamin D-dependent rickets type 1**	↑	↓↓	↑	↓↓	↑	↑↓	↓↓	=↓
**Vitamin D-dependent rickets type 2**	↑	↓↓	↑	↓↓	↑	=↑↓	↑↑	=↓
**Drugs inhibiting mineralization**	=↑	=	=	=	=	=	=	=
**Hypophosphatasia**	↓↓	=	=	=	=	=	=	=
**FGF23-unrelated hypophosphatemic disorders**	↑	↓↓	=↑	=	=↑	=	=↑	=↓
**FGF23-related hypophosphatemic disorders**	↑	↓↓	↓↓	=	=↑	=	↓	↑↑

Legend: ALP: serum alkaline phosphatase; Ca: serum calcium; Pi: serum inorganic phosphate; TmP/GFR: renal tubular reabsorption of phosphate; PTH: parathyroid hormone; 25(OH)D: 25 hydroxyvitamin D; 1,25(OH)_2_D: 1,25 di-hydroxyvitamin D; FGF23: fibroblast growth factor 23.

**Table 3:** Genetically determined forms of osteomalacia due to (A) FGF23-mediated hypophosphatemic disorders; (B) non-FGF23 mediated hypophosphatemic disorders; (C) altered vitamin D metabolites or signaling.

Disease	Gene	OMIM Phenotype Number	OMIM Gene Number	Inheritance
**A**. **FGF23-mediated Hypophosphatemic Disorders**				
X-linked dominant hypophosphatemic rickets/osteomalacia (XLH)	*PHEX*	# 307800	*300550*	XLD
Autosomal dominant hypophosphatemic rickets/osteomalacia (ADRH)	*FGF23*	# 193100	*605380*	AD
Autosomal recessive hypophosphatemic rickets/osteomalacia 1 (ARHR1)	*DMP1*	# 241520	*600980*	AR
Autosomal recessive hypophosphatemic rickets/osteomalacia 2 (ARHR2)	*ENPP1*	# 613312	*173335*	AR
Hypophosphatemic disease with dental anomalies and ectopic calcification	*FAM20C*	# 259775	*611061*	AR
Hereditary hypophosphatemic rickets with hypercalciuria (HHRH)	*SLC34A3*	# 241530	*609826*	AR
McCune–Albright syndrome/fibrous dysplasia (MAS)	*GNAS*	# 174800	*139320*	(postzygotic somatic mutations)
Cutaneous skeletal hypophosphatemia syndrome	*NRAS* *HRAS*	# 162900	*164790* *190020*	(somatic mutations)
**B**. **Non-FGF23-mediated Hypophosphatemic Disorders**				
Fanconi renotubular syndrome 1 (FRTS1)	*-*	# 134600	*-*	AD
Fanconi renotubular syndrome 2 (FRTS2)	*SLC34A1*	# 613388	*182309*	AR
Fanconi renotubular syndrome 3 (FRTS3)	*EHHADH*	# 615605	*607037*	AD
Fanconi renotubular syndrome 4 (FRTS4)	*HNF4A*	# 616026	*600281*	AD
Dent’s disease 1	*CLCN5*	# 300009	*300008*	XLR
Dent’s disease 2	*OCRL*	# 300555	*300535*	XLR
Lowe syndrome	*OCRL*	# 309000	*300535*	XLR
**C**. **Altered Vitamin D Metabolites or VDR Signaling**				
Vitamin D-dependent rickets/osteomalacia type 1A (VDDR1A)	*CYP27B1*	# 264700	*609506*	AR
Vitamin D-dependent rickets/osteomalacia type 1B (VDDR1B)	*CYP2R1*	# 600081	*608713*	AR
Vitamin D-dependent rickets/osteomalacia type 2A (VDDR2A)	*VDR*	# 277440	*601769*	AR
Vitamin D-dependent rickets/osteomalacia type 2B (VDDR2B)	*-*	# 600785	*-*	AD

Legend: AR = autosomal recessive; AD = autosomal dominant; XLD = X-linked dominant; XLR = X linked recessive.

## Data Availability

Not applicable.
